# The actions of neurotensin in rat bladder detrusor contractility

**DOI:** 10.1038/srep11192

**Published:** 2015-06-08

**Authors:** Xingyou Dong, Xinyu Bai, Jiang Zhao, Liang Wang, Qingqing Wang, Longkun Li

**Affiliations:** 1 Department of Urology, Second Affiliated Hospital, Third Military Medical University, Chongqing 400037, China.

## Abstract

This study assessed the expression, distribution and function of neurotensin (NTs) and two main neurotensin receptors (NTSR), NTSR1 and NTSR2 in normal rat urinary bladders. NTs is primarily located in the suburothelium and the interstitium of smooth muscle bundles. The NTSR1 and NTSR2 receptor subtypes are found to co-localize with smooth muscle cells (SMCs). NTs not only can directly act on bladder SMCs to induce intracellular calcium mobilization by activating the phospholipase C/inositol triphosphate (PLC/IP3) pathway, promoting extracellular calcium influx through a non-selective cation channels, but may be also involved in the modulation of the cholinergic system. Nowadays, the selective antimuscarinic drugs (solifenacin) and the selective beta 3-adrenergic agonist (mirabegron) are used as the first-line pharmacotherapy for overactive bladder (OAB), but without satisfactory treatment benefits in some patients. This study provided evidence suggesting that bladder NTs may play an important role in the regulation of micturition. Further research is needed to investigate the effects of NTs on bladder contractility and the underlying mechanism, which might reveal that the administration of NTSR antagonists can potentially relieve the symptoms of OAB by coordination with antimuscarinic pharmacotherapy.

Neurotensin (NTs), a 13-amino acid peptide, was originally isolated from calf hypothalamus by Carraway and Leeman in 1973^1^. NTs is widely expressed in the mammalian central nervous system (CNS) and functions in the dual roles of neurotransmitter and neuromodulator to contribute to a variety of physiological functions and pathological processes^2^. NTs is not only involved in the regulation of dopaminergic transmission and opioid-independent antinociception, hypothermia, analgesia, and pituitary hormone secretion, but it may also be related to Parkinson’s disease and schizophrenia^3^. These effects of NTs are exerted by two subtypes of neurotensin receptor (NTSR). The C-terminal 8–13 sequence of NTs is responsible for binding to and activating the NTSR1 and NTSR2. The high-affinity receptor NTSR1 and low-affinity receptor NTSR2 were first cloned by Tanaka K and Chalon P^4^, and subsequent studies have revealed that both of them belong to the G protein-coupled receptors (GPCRs), whose physiological functions have been well studied. However, two other NTSRs, NTSR3 and NTSR4, which are not well known, belong to the sortilin and sorla receptor superfamilies, respectively^5^.

NTs has also been the focus of many original studies on physiological function and peripheral regulation. In the cardiovascular system, NTs has a variety of biological effects, including the release of prostacyclin and histamine^6^, the regulation of cardiovascular contraction^7^ and cardio-respiratory effects^8^, influence blood pressure and heart rate^9^. In the digestive system, NTs works as an endocrine agent that is released into the blood from the entero-endocrine N cells in response to various biological stimuli. NTs plays an important role in some physiological actions, including the stimulation of gastric acid, pancreatic and biliary secretion to facilitate the absorption of fatty acids^10^. New ideas and evidences have emerged regarding the role of NTs in the contraction and pacemaker currents of the small intestine. NTs induces contraction of the small intestine smooth muscle in a concentration-dependent manner via its action on the NTSR1 of SMCs and interstitial cells of Cajal (ICCs)^11^,^12^. In addition, the relationship between NTs and cholinergic systems has been documented in several research articles. Both endogenous and microinjected NTs exert excitatory effects on cholinergic neurons. In the rat basal forebrain and magnocellularis nucleus, NTSR1 are located in the cell bodies of cholinergic neurons^13^,^14^. In the cerebral cortex and basal forebrain, NTs evoke endogenous acetylcholine (ACh) release from cholinergic neurons^15^,^16^.

Nevertheless, there are no studies of NTs in the urinary bladder, and its possible role and mechanism are unclear. Overactive bladder (OAB) is a syndrome that is defined as urgency, with or without urinary incontinence, usually associated with frequency and nocturia, which severely impacts the quality of life of patients. Antimuscarinic drugs are currently the first-line pharmacotherapy for OAB; however, more than 60% of patients eventually discontinue this treatment regimen because these drugs do not produce the expected treatment benefits^17^,^18^. Whether NTs is involved in the regulation of detrusor contraction and/or the cholinergic system is unknown. In this study, we characterized the expression and localization of NTs, NTSR1 and NTSR2 in mature rat urinary bladders by using primers and antibodies for NTs and NTSR. Furthermore, several pharmacological agents were applied in functional measurements to ascertain the influences of NTs on the bladder detrusor and cholinergic systems. These results provide the first evidence that NTs may play an important role in the modulation of bladder detrusor contractility.

## Results

### Expression of NTs, NTSR1 and NTSR2 in rat bladder

**Figure 1:**
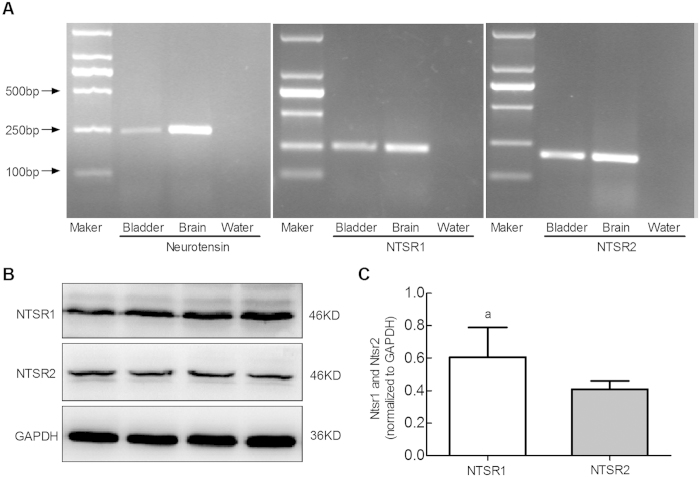
NTs and NTSR mRNA and protein expression in the rat bladder. The mRNAs for NTs and the two main subtypes of NTSR, NTSR1 and NTSR2, were detected in the rat bladder. Brain tissue and water were used as the positive and negative controls respectively (**A**). The identities of all products were confirmed by sequencing and reverse PubMed BLAST analyses. Western blots of the tissue lysates were probed with anti-NTSR1, and anti-NTSR2 and anti-GAPDH served as an endogenous control. The molecular weight of the NTSR was 46 kDa (**B**). Semiquantitative analysis of the NTSR subtypes. The expression level of NTSR1 was higher than that of NTSR2 ((**C**), ^a^P < 0.001, paired-sample t-test, four rats).

The reverse transcription-polymerase chain reaction results indicated that the mRNA of NTs (260 bp) and the two primary neurotensin receptors, NTSR1 (253 bp) and NTSR2 (198 bp), exist in rat bladders (Fig. 1A). Western blotting analysis revealed specific bands at 46 KDa for NTSR1 and 45 KDa for NTSR2 (Fig. 1B). The specificity of NTSR antibodies was identified by *Sigma-Aldrich* via performing the preabsorption control with western blotting and immunofluorescence (NTSR1: http://www.sigmaaldrich.com/catalog/product/sigma/sab4300718?lang=zh&region=CN; NTSR2: http://www.sigmaaldrich.com/catalog/product/sigma/sab4501016?lang=zh&region=CN). The expression level of NTSR1 relative to GAPDH may be higher than that of NTSR2 (Fig. 1C, 0.61 ± 0.18 vs. 0.41 ± 0.05, n = 6, P < 0.05).

### Distribution and colocalization of NTs, NTSR1, and NTSR2

**Figure 2:**
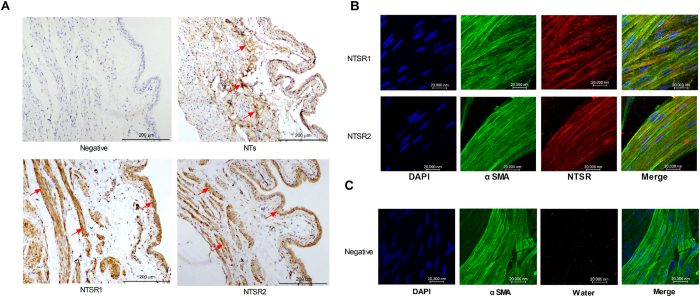
Location of NTs and the NTSR subtypes in the rat bladder tissue. The distribution of NTs and NTSR1 and NTSR2 was verified with immunohistochemistry. The red arrow indicates immunoreactive tissues/cells in the rat bladder. Compared with the negative control, NTs immunoreactive tissues mainly existed in the suburothelium and among the detrusor bundles. NTSR1 and NTSR2 immunoreactive cells mainly include smooth muscle cells and urothelial cells. Furthermore, the immunofluorescence results revealed that NTSR1 and NTSR2 co-labeled with the smooth muscle cells (**A**). Co-labeled immunofluorescence of NTSR and smooth muscle α-actin is revealed in Fig. 2B: 1, Nuclei counterstained with DAPI (blue); 2, anti-α-actin (green); 3, anti-NTSR (red); 4, merged image showing the co-localization of NTSR and SMCs. The negative control was incubated with water instead of anti-NTSR to exclude non-specific staining (**C**).

Next, we analyzed the distribution of NTs, NTSR1, and NTSR2 using the immunohistochemistry method (Fig. 2A). We observed that immunoreactive stained NTs are mostly present in the suburothelium region and partially distributed among smooth muscle bundles, but it is not clear whether NTs colocalize with nerve bundles, myofibroblast cells or interstitial cells, whereas NTSR1 and NTSR2 immunoreactive staining mainly occurs in the smooth muscle layer and urothelium compared with the negative control. Furthermore, the immunofluorescence results revealed that NTSR1 and NTSR2 were co-labeled with the smooth muscle cells (Fig. 2B). All immunoreactive staining results were compared with the negative control (Fig. 2C, replacing the primary antibodies with distilled water).

### Effects of NTs on the micturition process

**Figure 3:**
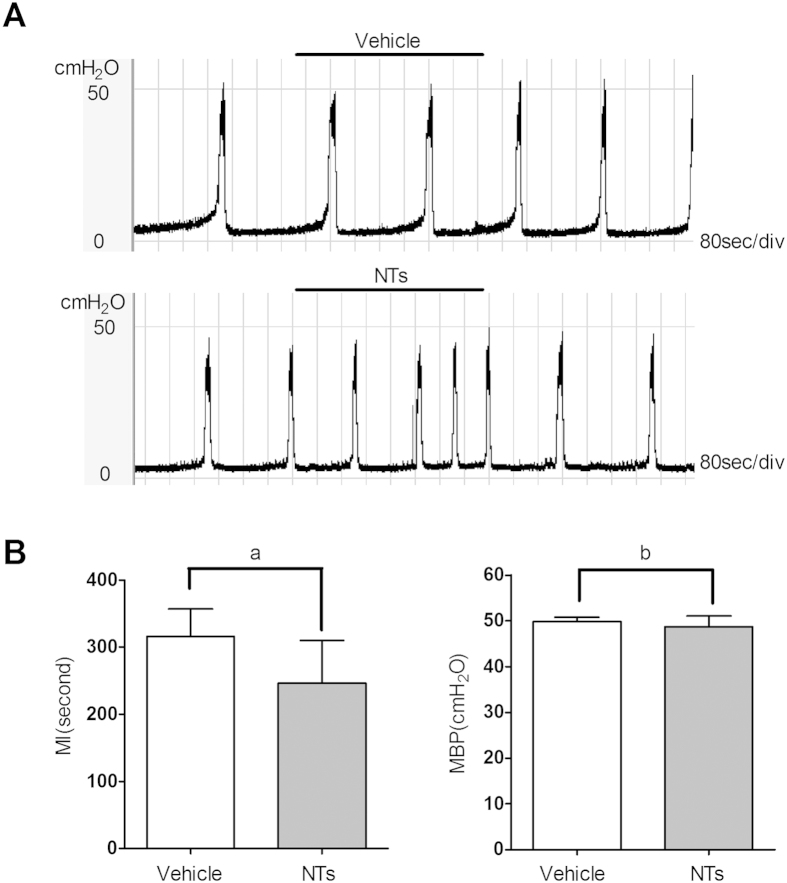
NTs-induced changes in bladder voiding. NTs-induced changes in bladder micturition were detected with rat cystometry. Infusion of NTs in rat bladder lumen can influence the MI but not the MBP (**A**). Compared with saline vehicle, NTs significantly decreased the MI but had no influence on MBP (B, ^a^P = 0.001, ^b^P = 0.100, independent-sample t-test, 6 rats).

**Figure 4:**
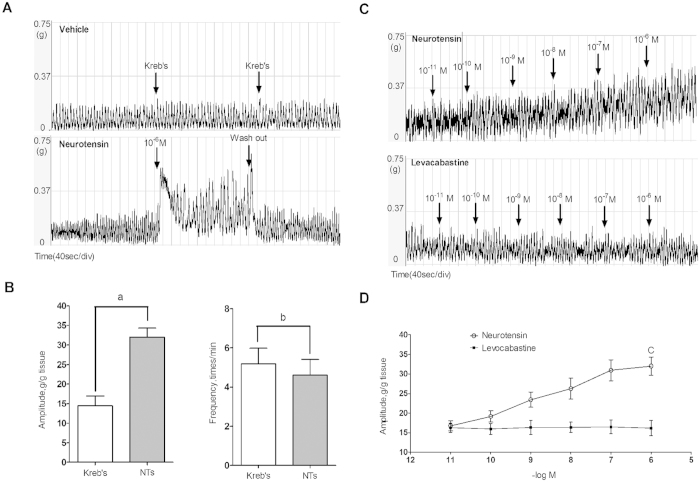
The effects of NTs and levocabastine on the spontaneous contraction of the detrusor muscle strips. Tension recordings revealed the effects of NTs on the contractile activities of the rat detrusor strips (**A**). Compared with negative control, NTs (1 μM) can increase the amplitude and decrease the frequency (**B**), ^a^P < 0.001, ^b^P < 0.01, independent-sample t-test, 10 muscle strips) of detrusor strip contraction, which recovered after washing. The dose-response effects of NTs (10^–11^ to 10^–6^ M) and levocabastine (10^–11^ to 10^–6^ M) were also measured (**C**). Gradual increases in amplitude were found in the NTs group (**D**), ^c^P < 0.001 indicates that the concentration 10^–6^ M had more significant effects than other concentrations, one-way ANOVA, seven muscle strips) but not for levocabastine (P = 0.115, one-way ANOVA, six muscle strips).

To analyze the effects of NTs on bladder micturition, cystometry was performed on unconscious rats, and a continuous pressure curve was recorded after intravesical administration of saline and NTs (Figs 3A and 4B). Infusion with NTs (1 μM) decreased the micturition interval (MI) (316.00 ± 41.25 vs. 246.53 ± 63.44 seconds, P < 0.01) (Fig. 3C), but it had no influence on the maximum bladder pressure (MBP) (49.84 ± 1.01 vs. 48.88 ± 2.20 cm H_2_O, P = 0.10) (Fig. 3D) compared with the saline vehicle. The changes in MI suggested that NTs can mediate bladder micturition by influencing voiding frequency.

### Associated effects among NTs and the cholinergic system in detrusor strip contraction

**Figure 5:**
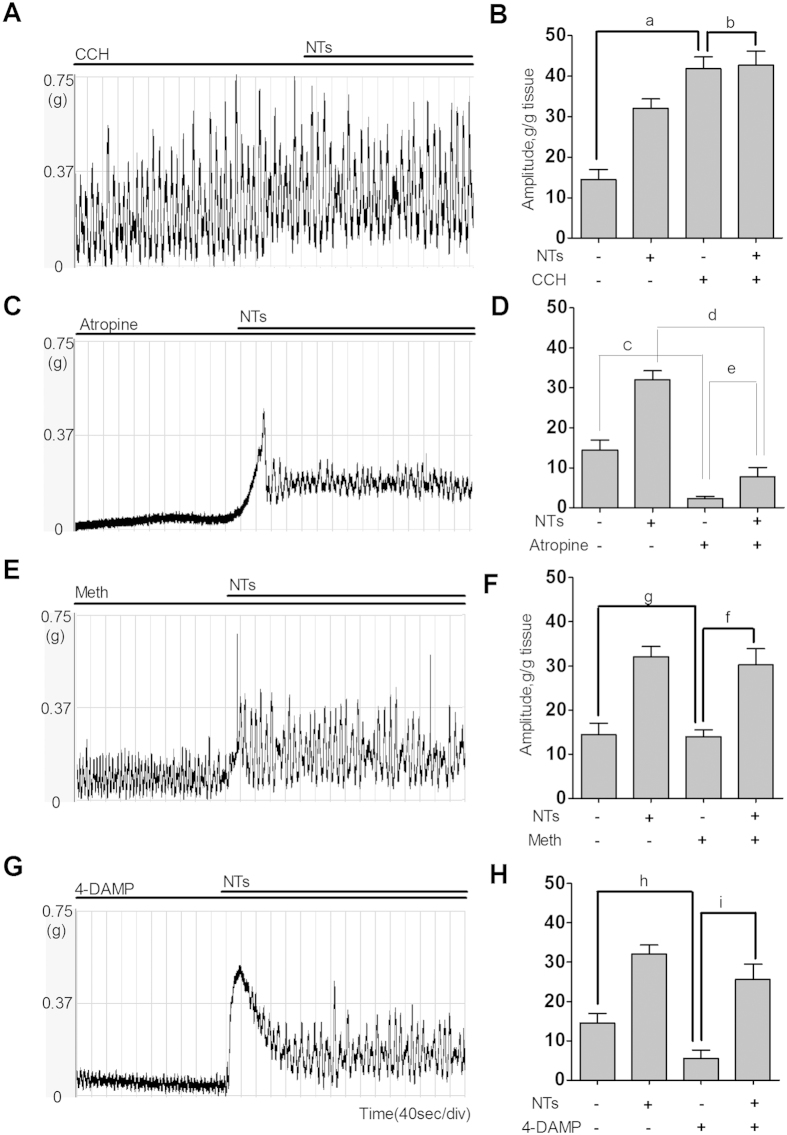
The relationship between NTs and the cholinergic system in tension recording. Treatment with CCH (0.1 μM) can increase contractile amplitude (**A**,**B**, ^a^P < 0.001), whereas NTs did not further increase the effects induced by CCH (^b^P = 0.072, one-way ANOVA, eight muscle strips). Therefore, upon pretreatment with atropine (1 μM), the spontaneous and NTs-induced contractions were drastically inhibited (**C**,**D**, ^c^P < 0.001, ^d^P < 0.001). However, NTs reversed the inhibition of contraction amplitudes induced by atropine (^e^P < 0.001). Similarly, NTs increased contraction amplitude after pretreatment with METH (1 μM) (**E**,**F**, ^f^P < 0.001). METH did not significantly inhibit the amplitude of spontaneous contraction (^g^P = 0.094, one-way ANOVA). However, 4-DAMP inhibited the amplitude of spontaneous contraction (**G**,**H**, ^h^P < 0.001), and NTs reversed the inhibition. (^i^P < 0.001, one-way ANOVA).

Given that NTs is involved in the regulation of bladder voiding, we tested the effect of NTs on detrusor strip contraction. Using isometric tension recordings, NTs was added to a bath to examine its effects on isolated detrusor strip contraction (Fig. 4A). The administration of vehicle Kreb’s solution had no influence on detrusor strip contraction, whereas treatment with NTs (1 μM) significantly increased the amplitude (32.02 ± 2.35 vs. 14.45 ± 2.53 g/g tissue, P < 0.001) and decreased the frequency (4.60 ± 0.81 vs. 5.18 ± 0.80 times/min, P < 0.01) of contraction (Fig. 4B). Concentration-response effects were also detected following the administration of a gradient concentration of NTs and levocabastine (10^–11^ M to 10^–6^ M) (Fig. 4C). Increasing the NTs concentration resulted in enhanced contractility of the muscle strips that was primarily manifested as increases in amplitude, but different concentrations of levocabastine had no effect on detrusor contraction (Fig. 4D). In an experiment examining the relationship between NTs and the cholinergic system, NTs increased the contraction amplitude after pretreatment with 0.1 μM carbachol (CCH), but with no significant differences (42.75 ± 3.41 vs. 41.85 ± 2.88 g/g tissue, P = 0.072) (Fig. 5B), When the detrusor strips werepretreated with atropine, 4-DAMP, and methoctramine (METH), the spontaneous phasic activities were inhibited to a different degree by atropine (2.32 ± 0.58 vs. 14.45 ± 2.53, P < 0.001) or 4-DAMP (8.64 ± 2.25 vs. 14.45 ± 2.53 g/g tissue, P < 0.001), but not by METH (13.92 ± 1.61 vs. 14.45 ± 2.53 g/g tissue, P = 0.094), NTs partially reversed the inhibition by atropine (7.84 ± 2.23 vs. 2.32 ± 0.58 g/g tissue, P < 0.001) or 4-DAMP (25.63 ± 3.85 vs. 8.64 ± 2.25 g/g tissue, P < 0.001), and increased the contraction amplitude after pretreating with METH (31.10 ± 3.51 vs. 13.92 ± 1.61 g/g tissue, P < 0.001, Fig. 5D,F,H).

### NTs modulates the properties of intracellular calcium and the inward current of SMCs

**Figure 6:**
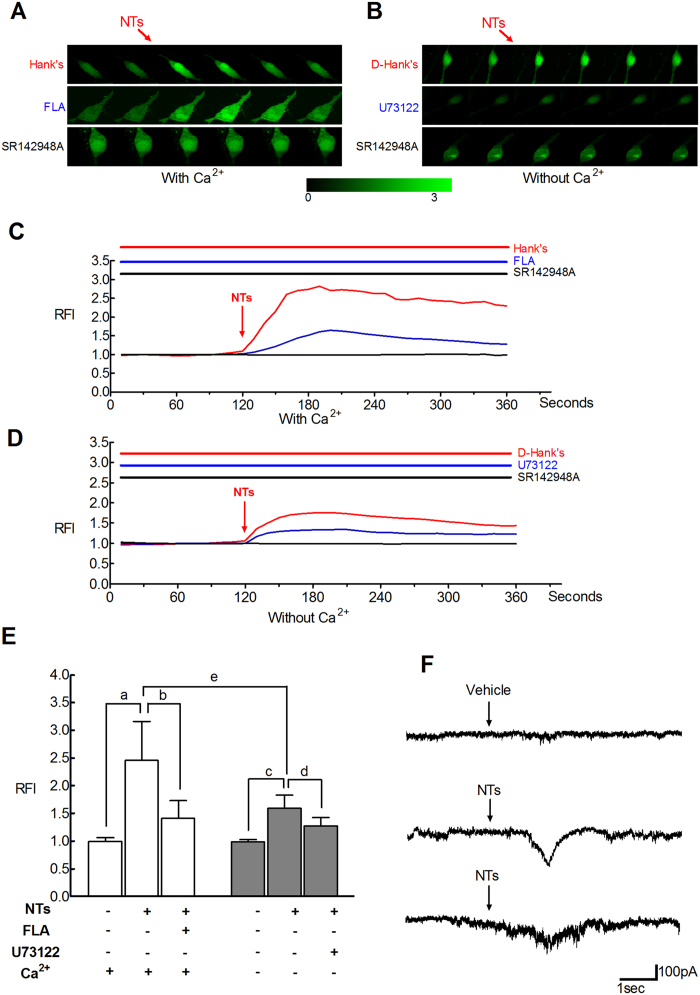
The effects of NTs on intracellular calcium concentration in SMCs. Every six pictures were chosen to represent the process of instantaneous [Ca^2+^]_i_ changes in the SMCs after treatment with pharmacological agents (**A**,**B**). The real-time relative fluorescence intensities (F1/F0) are shown as continuous plots (**C**,**D**). In a calcium solution, application with NTs increased [Ca^2+^]_i_ (E, ^a^P < 0.001), which was drastically abolished by pretreatment with SR142948A (P < 0.001) and partly inhibited by FLA (^b^P < 0.001). In a calcium-free solution, NT administration also increased [Ca^2+^]_i_ (^e^P < 0.001), and the increasing effects of NTs on calcium concentration with calcium solution was higher than that in the calcium-free solution (^c^P < 0.001). Pretreatment with SR142948A and U73122 can also completely abolish or partially inhibit the effects (^d^P < 0.001). In the whole cell patch clamp experiment, NTs triggered an inward current, from 40 pA to 180 pA, but the vehicle had no influence on the cell current.

Under a voltage clamp at a holding potential of −60 mV, a large inward current was evoked in response to 1 μM NTs, whereas the vehicle had no influence on the current (P < 0.01). The peak amplitude of the inward current varied from 40 pA to 180 pA in different cells (n = 5), and the duration time varied from 1.2 seconds to 4.4 seconds (Fig. 6F). To elucidate the mechanism of inward current induced by NTs, we measured the calcium ion activity using primary isolated SMCs. A calcium ion probe, Fluo-4 AM, was used to stain intracellular calcium ions. We detected the effects of NTs in two different incubation solutions, Hank’s (with Ca^2+^) and D-Hank’s (without Ca^2+^) solution. SMCs images were acquired at a rate of six frames per minute, and six of them were chosen as representative of the changing relative fluorescence intensities (RFI = F1/F0, where F1 is the real-time fluorescence intensity, and F0 is the baseline fluorescence intensity before administration) (Fig. 6A,B). According to the average fluorescence value, we described the entire process via plots of single curves based on the recorded RFIs (Fig. 6C,D). Compared with the vehicle, NTs significantly increased intracellular calcium ion concentration ([Ca^2+^]i) regardless of whether samples were incubated with Hank’s (2.45 ± 0.71 vs. 1.00 ± 0.06, P < 0.001) or D-Hank’s solution (1.60 ± 0.23 vs. 1.00 ± 0.06, P < 0.001). In addition, the increased concentration of calcium ions in Hank’s solution was higher than that in D-Hank’s solution (2.45 ± 0.71 vs. 1.60 ± 0.23, P < 0.001). The influences of NTs on [Ca^2+^]i were completely blocked by pretreatment with NTSR antagonist, SR142948A (1 μM, 1.00 ± 0.07 vs. 2.45 ± 0.71, P < 0.001; 1.00 ± 0.04 vs. 1.60 ± 0.23, P < 0.001). These results strongly suggested that NTs could influence detrusor myocyte depolarizing potential by elevating the [Ca^2+^]i of SMCs. Therefore, two special pharmacological agents, flufenamic acid (FLA), a non-selective cation channel blocker, and U73122, a PLC/IP3 signal pathway inhibitor, were used to study the potential mechanism of the changed [Ca^2+^]i. Analogously, the effects of NTs were partly blocked (1.41 ± 0.33 vs. 2.45 ± 0.71, P < 0.001) by FLA (100 μM) in Hank’s solution and by U73122 (1 μM) in D-Hank’s solution (1.27 ± 0.16 vs. 1.60 ± 0.23, P < 0.001) (Fig. 6E).

## Discussion

The traditional view of the regulation of urinary bladder function focus on detrusor smooth muscle cells and their nerve innervation; however, the urothelium and the underlying suburothelium have recently emerged as a combined regulator of bladder activity^19^. In response to stimuli, including mechanical distension and the presence of specific molecules, the releasing factors, such as adenosine triphosphate, prostaglandins G, nerve growth factor, nitric oxide and ACh, modulate detrusor muscle contraction^20^ and sensory nerve activity^21^. However, whether other bioactive molecules are released from the urothelium/suburothelium unit is not clear.

We detected the expression and functional characteristics of NTs and NTSR in the rat urinary bladder for the first time. NTs was detected at the mRNA and protein levels using RT-PCR and immunohistochemistry, respectively. The positive sites for NTs protein were predominantly distributed in the suburothelium and among the detrusor bundles. This result suggested that NTs may be released from the urinary bladder in response to different stimuli to exert corresponding biological effects. In the gastrointestinal tract, an increasing number of biological effects have been attributed to NTs discovery. The evidence indicates that NTs released from entero-endocrine N cells^22^ is involved in slow wave regulation in the gastrointestinal tract and promotes the peristaltic activity of the intestine by acting on NTSR1^11^,^23^. Although the bladder, which is an urinary organ regulating the storage and elimination of urine, has a physiological function different from the gastrointestinal tract, the urinary tract and gastrointestinal tract share a common mechanism in generating spontaneous myogenic contractions. This activity can occur in the absence of any neural input, implying the presence of an intrinsic pacemaker. The ureter and bladder muscle strips also show electrical slow waves similar to those of the gastrointestinal tract because of the presence of interstitial cells that are similar to ICCs in the gastrointestinal tract^24^.

In this study, we confirmed the presence of NTSR1 and NTSR2 mRNA and protein in rat bladders and investigated the localization of NTSR. The existence of NTSR in smooth muscle cells most likely provides a structural basis to ensure the interaction of NTs and SMCs. The NTs-NTSR pathway may play a receptor-mediated role in bladder smooth muscle contraction. The locolization of NTs and NTSR in the bladder indicates their mutual actions. However, because of their different location in different bladdr tissue layers, their participation in the mediation of excitation requires further investigation. In our immunofluorescence labeling experiment, although the results showed the specific staining of NTs in smooth muscle cells, the antibodies for GPCRs are notorious for high levels of non-specific binding to closely related receptors, and thus more research is needed to verify the distribution of NTs and NTSR.

To determine a possible physiological role for NTs in the control of bladder voiding, filling cystometry experiments were conducted on anaesthetized rats. The instillation of NTs (1 μM) into the bladder lumen decreased the micturition interval. The effects of NTs on micturition may be related to the existence of NTSR in the urothelium and detrusor myocytes. However, overwhelming evidence suggests that urothelial cells are involved in bladder sensory function, which is the important part of the micturition reflex^25^. Therefore whether exogenous NTs affect bladder excitation via the NTSR in urothelial cells requires further investigation. As nerve fibers and detrusor myocytes are the likely functional targets, we studied the effects of NTs on the contractile activity of isolated bladder detrusor muscle strips of rats and the possible mechanism involved. We found that NTs can significantly increase the amplitude of strip contraction and slightly decrease the contraction frequency, whereas the NTSR2 agonist, levocabastine at different gradient concentrations (10^–11^ to 10^–6^ M), had no effect on detrusor contraction. These results might potentially suggest a more tight relationship between detrusor contraction and NTSR1 than NTSR2. A concentration-response effect of NTs was also detected in the isometric contraction experiment. With an increase in the NTs concentration (10^–11^ to 10^–6^ M), the contraction amplitude gradually increased. The high-affinity receptor of NTs, NTSR1, similar to other bladder micturition-related functional receptors, such as muscarinic, adrenergic and purinergic receptors, belongs to the family of GPCRs^25^. Specific ligands can bind to these receptors to participate in the regulation of bladder detrusor contraction via activation of the calcium ion-relevant pathway. Calcium ions within cells act as a type of message transport substance, with importance in cellular activities such as cell movement, cell proliferation, cell excitation and excitation-contraction coupling^26^. The contraction is initiated by an increase in cytosolic calcium.

Therefore, it is helpful for us to further understand the molecular mechanisms by measuring the effects of NTs on the intracellular calcium concentration of SMCs. In a calcium solution (1.26 mM), NTs enhanced intracellular calcium concentration. This effect was partly inhibited by pretreatment with a non-selective cation channel blocker, FLA (100 μM), and completely abolished by SR142948A (10 μM). These results indicate that NTs can increase bladder excitability by evoking Ca^2+^ influx via non-selective cation channels on SMCs. The findings are further supported by an NTs-induced inward current in patch clamp experiments. Under a voltage clamp at –60 mV holding potential, the whole-cell inward currents of approximately 40 pA to 180 pA were induced in different detrusor myocytes. In a calcium-free solution, NTs also could promote intracellular Ca^2+^ mobilization, whereas this effect was partly suppressed in the presence of U73122 and was abolished by SR142948A. These results suggest that NTs could activate PLC-mediated IP3 production, thereby inducing IP3 receptor-mediated release of [Ca^2+^]i from the endoplasmic reticulum. This result, which is consistent with previous reports from the gastrointestinal tract^12^, provides strong support for the suggestion that non-selective cation channels and the activation of the PLC/IP3 pathway are essential for intracellular Ca^2+^ mobilization induced by NTs. Therefore, it can be speculated that the effects were achieved by inducing calcium ion influx mediated by non-selective cation channels and evoking the release of the calcium pool in the endoplasmic reticulum by activating the PLC/IP3 pathway to accelerate the process of myocyte depolarization.The parasympathetic nerve-regulated contraction of the urinary bladder is mainly due to the action of ACh via the activation of muscarinic M_2_ and M_3_ receptors. ACh binding to the M_3_ receptor can directly induce detrusor contraction. Conversely, the activation of muscarinic M_2_ receptors was demonstrated to constrain β-adrenergic receptor-mediated relaxation of the rat urinary bladder^27^. Conservative treatment options for OAB include lifestyle modification, pelvic floor exercises and medication. Although many researches have focused on the use of anticholinergic medications to treat OAB, the effectiveness is still not satisfactory^28^. Anticholinergics are considered to be the most effective drugs at present. Solifenacin, a selective M_3_ receptor antagonist, is easily acceptable to OAB patients because of its high selectivity and lower level of adverse effects, but it still cannot completely alleviate the suffering of patients^29^. Thus, continuing research on anticholinergic drugs is still very necessary. The relationship between NTs and cholinergic systems has been shown in the CNS by different researchers. Thus, we asked whether the effect of NTs is also linked to the cholinergic system, and attempted to verify the role of NTs in the modulation of the cholinergic system via a detrusor strip isometric contraction experiment.

The cholinergic agonist CCH and the muscarinic blockers, M_2_ and M_3_ acceptor antagonists, atropine, METH and 4-DAMP, respectively, were used to elucidate the effects of NTs and cholinergic system on detrusor muscle contraction. After pretreatment with CCH, NTs further increased the detrusor muscle strip contraction induced by CCH, although with no statistical significance (P = 0.072). It is not clear that the increase in the contraction amplitude was caused by the interaction of NTs and the cholinergic system or by the simple additive effects of NTs and CCH. NTs can partially reverse the inhibition of detrusor contraction by atropine or 4-DAMP, and increase that with METH, suggesting an improvement of detrusor contraction under several conditions, and might be a potential strategy for treatment. Over the last 10 years, emerging therapeutic drugs, such as botulinum toxin^30^ and mirabegron^28^, have been used for the treatment of OAB when standard treatments, such as bladder training and oral anticholinergic medication, have failed to provide relief. This research provides an experimental basis for studying the biological function of NTs and the cholinergic system and provides a new perspective on OAB medication. Further studies are required to be performed to confirm whether NTs is truly involved in the regulation of the cholinergic system in the urinary bladder.

Moreover, numerous studies related to NTs have emphasized the effects of this molecule on inflammation and cancer in many organs. NTs and NTSR are involved in the growth, proliferation, invasion and metastatic processes of many malignant tumors, such as those of the lung, prostate, pancreas, and colon^31^,^32^. Whether alterations in the release and function of NTs are involved in the pathological mechanism of several types of bladder dysfunction, including OAB, neurogenic bladder and painful bladder syndrome, is still unknown.

Although one limitation of this study was a lack of verification of the function of NTSR2 and the significance of urothelial NTSR, based on the present findings, we can speculate that the function of the urothelium/suburothelium is more complex than previously thought. The signaling molecules released from the urothelium may be not limited to adenosine triphosphate, prostaglandins G, nerve growth factor, nitric oxide and ACh, but may also include NTs and other molecules of which we are currently unaware. Endogenous NTs, which may exist in the suburothelium, might participate in modulation of micturition reflex and detrusor myocyte excitation-contraction coupling via directly depolarizing activity, thus mediating the [Ca^2+^]i of smooth muscle cells, or via indirectly influencing the function of the cholinergic system regardless of the urothelium non-neuronal cholinergic system or cholinergic nerves. Further studies of the functional mechanisms of the NTs-NTSR pathway are needed to fully elucidate the physiological and pathological roles of NTs in the bladder.

## Methods

### Animals

The methods were carried out accordance with the approved guidelines, and were approved by the Research Council and Animal Care and Use Committee of the Third Military Medical University, China (approval no. SYXK20070002). Fifty-two adult female Sprague-Dawley rats weighing 200–250 g were used in our experiments, housed in a room maintained at a constant temperature of 22–25 °C under a 12 h light/12 h dark cycle. All efforts were made to minimize animal suffering and the number of animals used. After the experiments were completed, all animals were killed with an intravenous overdose of pentobarbital sodium.

### Reverse-transcription PCR analyses of neurotensin and two receptor subtypes

A total of 100 mg of bladder tissue was mechanically homogenized and collected in 1 mL of ice-cold Trizol reagent (Tbdscience, Tianjin, China). The total RNA was extracted with chloroform, precipitated with isopropyl alcohol, and dissolved in RNAse-free water. Two-step reverse transcription-polymerase chain reaction (RT-PCR) was performed using a PrimeScript RT reagent kit (TaKaRa Bio, Tokyo, Japan) and 2 × Taq MasterMix (CWbio, Beijing, China) according to the manufacturer’s protocol. Reaction conditions were optimized for each of the genes (i.e., NTs, NTSR1, and NTSR2) by adjusting the annealing temperature (56 °C, 58 °C and 59 °C, respectively) and cycle numbers (30). Brain tissue and distilled water were, used as the positive and negative controls, respectively. The PCR products were verified by sequencing and the PubMed BLAST tool. All PCR primers were designed and synthesized by Sangon Biotech (Shanghai, China). The primer sequences and lengths of the PCR products were as follows: NTs, forward: 5’-GCGGGAGAAATGCGTGATG-3’, reverse: 5’-TAGGGCCTTCTGGTTTTATTCT-3’, 260 bp; NTSR1, forward: 5’-GGGCACACACAACGGTTTAGAG-3’, reverse: 5’-GATGGCGGAGCTGACGTAGAAGA-3’, 253 bp; and NTSR2, forward: 5’-GCGCCCAGGTTCTCAGAGC-3’, reverse: 5’-CGGCGTTGTAGAGGATTGGGG-3’, 198 bp.

### Detecting protein expression of neurotensin receptors by western blotting

Six SD rat urinary bladders were dissected. The extracted proteins were used for western blotting as we reported previously. Approximately 50 mg of total protein was separated on 12% sodium dodecyl sulfate polyacrylamide gels and transferred to polyvinylidene fluoride membranes. After blocking nonspecific binding with 5% bovine serum albumin, the blots were incubated with the primary antibodies (rabbit polyclonal anti-NTSR1 and NTSR2 were purchased from *Sigma-Aldrich*, St. Louis, USA, the cat numbers of NTSR1 and NTSR2 are SAB4300718 and SAB4501016, respectively; dilutions of 1:600 and 1:700 for NTSR1 and NTSR2) overnight at 4 °C. After incubation with HRP-conjugated goat anti-rabbit IgG (1:3000, Zhongshan Inc., Beijing, China), the protein band images were collected, and the relative optical densities (R.O.Ds) were analyzed with a ChemiDoc XRS+Image System (Bio-Rad Laboratories).

### Labeling neurotensin and neurotensin receptors by immunohistochemistry

The bladder specimens were preserved in a 2.5% glutaraldehyde-polyoxymethylene solution, embedded in paraffin and cut into 5-mm-thick sections. After blocking with rabbit serum at room temperature for 15 min, the sections were incubated in rabbit anti-rat NTs polyclone antibody (purchased from Genetex Inc., Texas, America; Cat number is GTX37368; at a dilution of 1:300) and rabbit anti-NTSR polyclone antibodies (dilutions of 1:200) overnight at 4 °C. After rinsing the sections with PBS, goat anti-rabbit-conjugated fluoresceinisothiocyanate secondary antibodies (Zhongshan Inc., Beijing, China) were applied to the sections, which were incubated at 37 °C for 30 minutes. After incubation with streptavidin/HRP at 37 °C for 30 minutes, the sections were colored with 3,3-diaminobenzidin (DAB), stained with hematoxylin and dehydrated, cleared and mounted in neutral gum. An identical procedure was used for the control group except that the rabbit anti-rat NTs was replaced with distilled water.

### Colocalization of NTSR and SMCs by immunofluorescence staining

The bladders of eight rats were fixed in 4% paraformaldehyde for 12 hours at 4 °C, and the mucosa was carefully removed. The thin sheet samples of the muscle bundles were incubated in 1% BSA for 2 hours and permeated in 0.3% Triton X-100 for 10 minutes. The primary polyclonal antibodies were incubated overnight at 4 °C. The NTSR primary antibody and smooth muscle α-actin antibody (Santa, California, America) were used at a dilution of 1:200 and 1:250, respectively. The secondary fluorescent antibodies conjugated with tetraethyl rhodamine isothiocyanate and fluorescein isothiocyanate (Molecular Probes, Eugene, OR) were incubated to identify positive labeling at 1:400 for 2 hours. The samples were incubated with 4’,6-diamidino-2-phenylindole (DAPI, Sigma-Aldrich, St. Louis, MO) at 1:10 for 10 minutes to stain the nuclei. After the samples were mounted on microscope slides, superimposed pictures were captured with a confocal microscope (Leica, Solms, Germany).

### Cystometric evaluation in unconscious rats

Experiments were performed on intraperitoneal urethane-anesthetized rats (n = 5, 1 mg/kg body weight). The catheter (PE50) was introduced into the bladder through the urethral opening and connected to a three-way pipe fixed with a pressure transducer and an infusion pump (AVI 270, 3M, Minnesota, USA). The pressure transducer was connected to a multi-channel signal acquisition system (RM6480C, Chengyi, Chengdu, China) that could amplify and reveal tension signals on a computer. Room temperature saline or 1 μM NTs dissolved in saline was infused at a rate of 0.1 ml/min. The following cystometric parameters were investigated: maximum bladder pressure and micturition interval.

### ***In vitro*** detrusor muscle strips tension measurement

The rat bladders were longitudinally cut into approximately 2 × 2× 6-mm strips. Each strip was suspended vertically between two curved hooks and placed into a 10-mL organ bath that was filled with Kreb’s solution and maintained at 37 °C, 95% O_2_ and 5% CO_2_. The upper hook was connected to a movable stretch transducer, and the lower hook was fixed to the bottom of the bath. After equilibrating for 30 minutes, the strip was pre-loaded at 0.75 g of initial stretch. The continuous dynamic curves were recorded with isometric force transducers (Chengyi Co., Chengdu, China) and visualized with RM6280C (Chengyi Co., Chengdu, China). Gradient concentrations of NTs (10^–11^–10^–6^ M) and levocabastine (LEVO), an NTSR2 agonist (10^–11^–10^–6^ M), were used to study the effects on muscle strip contraction. In addition, several pharmacological agents, including the cholinergic receptor agonist CCH (0.1 μM), the muscarinic blocker, atropine (1 μM), and the M_2_ and M_3_ receptor antagonists METH (1 μM) and 4-DAMP (1 μM) (all these agents were purchased from Sigma-Aldrich), were applied to identify the possible mechanism. The Kreb’s solution contained the following (in mmol/L): 119NaCl, 4.7 KCl, 1.2 KH_2_PO_4_, 1.2 MgSO_4_·7H_2_O, 25 NaHCO_3_, 2.5 CaCl_2_ and 11 glucose, adjusted to pH 7.4 with NaOH.

### Detecting inward currents induced by NTs using a whole cell patch clamp

Freshly dispersed bladder SMCs were transferred to the chamber for cell adherence and superfused with the following solutions (in mM): 152 NaCl, 5 KCl, 1 MgCl_2_, 2 CaCl_2_, 10 glucose, and 10 HEPES, adjusted to pH 7.4 with NaOH. The whole cell configuration of the patch clamp technique was used to record membrane currents (voltage clamp). Glass pipettes (4–6 mΩ) were filled with the following (in mM): 140 KCl, 2 MgCl_2_, 0.5 CaCl_2_, 2 ATP-Mg, 2 GTP-Li, 20 HEPES, and 5-ethylene glycol tetraacetic acid (EGTA), adjusted to pH 7.2 with KOH. The SMCs were maintained at a holding potential of −60 mV, which was close to the mean resting potential to detect the inward currents normalized to cell capacitance. NTs (1 μM) was applied to record the current responses in different SMCs. The data were recorded and analyzed using a pCLAMP10.2 (Axon Instruments, USA).

### Primary bladder smooth muscle cell isolation and intracellular Ca^2+^ concentration measurement using confocal microscopy

The smooth muscle cells were freshly dispersed for approximately 40 minutes at 37 °C in digestion solution containing the following (mg/mL): 2.0 type II collagenase, 2.0 BSA, and 2.0 trypsin inhibitor (all from Sigma-Aldrich, St. Louis, MO). After washing in Hank’s (with calcium) or D-Hank’s (without calcium) Balanced Salt Solution, the cells were filtered away from the tissue fragments with a 200-mesh cell strainer. Next, the cells were seeded onto a glass coverslip at the bottom of a recording chamber filled with Dulbecco’s modified Eagle’s medium with 10% fetal bovine serum (Sigma-Aldrich) and 1% penicillin-streptomycin at 37 °C, 95% O_2_ and 5% CO_2_. Eight hours later, the cells were washed with PBS solution and loaded with Fluo-4 AM (5 μM, Calcium Ion Probes) for 30 minutes at 37 °C. Ca^2+^ imaging experiments were captured at an emission wavelength of 488 nm. NTs (1 μM), the NTSR antagonist SR142948A (1 μM), the non-selective cation channels blocker FLA (100 μM) and the PLC signal pathway inhibitor U73122 (1 μM) (Sigma-Aldrich, St. Louis, MO) were used to examine the effects on real-time [Ca^2+^]i with laser confocal microscopy. The effects are presented as the relative fluorescence intensities RFI = F1/F0, where F1 is the real-time fluorescence intensity, and F0 is the baseline fluorescence intensity before administration.

### Statistical Analyses

The experimental data are presented as the mean ± SD. Student’s t-tests or one-way ANOVA were calculated using SPSS 16.0 software (SPSS Inc., Chicago, IL) for the comparisons. All statistical tests were two-tailed and differences with P values below 0.05 (P < 0.05) were considered to be statistically significant.

## Additional Information

**How to cite this article**: Dong, X. *et al.* The actions of neurotensin in rat bladder detrusor contractility. *Sci. Rep.* **5**, 11192; doi: 10.1038/srep11192 (2015).
